# UPLC-TOF-MS Characterization and Identification of Bioactive Iridoids in *Cornus mas* Fruit

**DOI:** 10.1155/2013/710972

**Published:** 2013-10-08

**Authors:** Shixin Deng, Brett J. West, C. Jarakae Jensen

**Affiliations:** Research and Development Department, Morinda Inc., 737 East, 1180 South, American Fork, UT 84003, USA

## Abstract

*Cornus mas* L. is indigenous to Europe and parts of Asia. Although *Cornus* is widely considered to be an iridoid rich genera, only two iridoids have been previously found in this plant. The lack of information on taxonomically and biologically active iridoids prompted us to develop and optimize an analytical method for characterization of additional phytochemicals in *C. mas* fruit. An ultra performance liquid chromatography (UPLC) coupled with photodiode array spectrophotometry (PDA) and electrospray time-of-flight mass spectrometry (ESI-TOF-MS) was employed and mass parameters were optimized. Identification was made by elucidating the mass spectral data and further confirmed by comparing retention times and UV spectra of target peaks with those of reference compounds. Primary DNA damage and antigenotoxicity tests in *E. coli* PQ37 were used to screen the iridoids for biological activity. As a result, ten phytochemicals were identified, including iridoids loganic acid, loganin, sweroside, and cornuside. Nine of these were reported for the first time from *C. mas* fruit. The iridoids did not induce SOS repair of DNA, indicating a lack of genotoxic activity in *E. coli* PQ37. However, loganin, sweroside, and cornuside did reduce the amount of DNA damage caused by 4-nitroquinoline 1-oxide, suggesting potential antigenotoxic activity.

## 1. Introduction

*Cornus mas* L. is commonly known as European cornelian cherry and belongs to the Cornaceae family. It is a tall deciduous shrub or small tree (3–6 m in height) that is indigenous to Europe and parts of Asia [1, 2]. The fruit is edible but is astringent when unripe. Traditionally, *C. mas* has been used for improving health conditions, such as bowel complaints, fever, and diarrhea [3–6]. Fresh European cornelian cherry fruits are often processed to produce drinks, syrups, and jams [7, 8]. 

Some investigations of the nutritional and phytochemical properties of European cornelian cherry fruit have been reported previously. The fruit is reported to contain 0.1–0.3% fat, 0.4% protein, 21.7% carbohydrate, 0.8% ash, 0.5% dietary fiber, 6.6–15.1% total sugar (fructose 33.1–43.1%, glucose 53.6–63.1%), and 4.22–9.96% reducing sugars [3, 9]. The fruit has pH 2.7–3.2 and contains at least 15 amino acids, including aspartic acid, glutamic acid, serine, histidine, glycine, threonine, arginine, alanine, tyrosine, valine, phenylalanine, isoleucine, leucine, lysine, and proline [9]. Minerals in *C. mas* fruit include copper (1.2 to 8.1 mg/kg dry weight), iron, zinc, phosphorus, potassium, calcium, magnesium, and sulphur [9]. The vitamin C content of the fruit is reported to be 16.4 to 38.5 mg/100 g [9]. 

*C. mas* fruit has been found to contain a wide range of phytochemicals, including tannins (131.51–601.2 mg/L), phenolics (29.76–74.83 mg/g dry), organic acids (4.6–7.4%), anthocyanin, fatty acids, and flavonoids [3, 4, 10–16]. Anthocyanins seem to have been a popular subject of previous *C. mas* research. Anthocyanins have been found in the range of 1.12 to 2.92 mg/g in different *C. mas* fruit sources [14, 16, 17]. Two studies from Du and Francis reported the presence of five anthocyanins in the fruit, namely, delphinidin 3-galactoside, cyanidin 3-galactoside, cyanidin 3-rhamnosylgalactoside, pelargonidin 3-galactoside, and pelargonidin 3-rhamnosylgalactoside [18, 19]. Cyanidin 3-glucoside, cyanidin 3-rutinoside, and pelargonidin 3-glucoside were identified by Tural and Koca [15], with pelargonidin 3-*O*-glucoside being the most abundant followed by cyanidin 3-*O*-*β*-D-galactoside. Pelargonidin 3-*O*-rutinoside was present only in trace amounts [17]. Delphinidin 3-*O*-*β*-galactopyranoside, cyanidin 3-*O*-*β*-galactopyranoside, and pelargonidin 3-*O*-*β*-galactopyranoside were also identified in *C. mas* fruit [3]. 

Eight flavonoids have been previously isolated from the methanolic extract of *C. mas *fruit. These include aromadendrin 7-*O*-*β*-D-glucoside, quercetin 3-*O*-*β*-D-xyloside, quercetin 3-*O*-*α*-L-rhamnoside, quercetin 3-*O*-rutinoside, quercetin 3-*O*-*β*-D galactoside, quercetin 3-*O*-*β*-D-glucose, quercetin 3-*O*-*β*-D-glucoside, and kaempferol 3-*O*-galactoside [17]. Fatty acids identified in the fruit include lauric acid, myristic acid, pentadecenoic acid, palmitic acid, palmitoleic acid, stearic acid, oleic acid, vaccenic acid, linoleic acid, linolenic acid, and *cis*-10,12-octadecadienoic acid [9]. 

*Cornus* is widely considered to be an iridoid rich genera. However, there is very little information regarding the occurrence of iridoids in *C. mas*. Only two iridoids, secologanin and loganic acid, have been previously reported to occur in *C. mas*, with only one being reported for the fruit [20–22]. The current investigation was prompted by a lack of information on taxonomically critical and biologically active iridoids in *C. mas* fruit. The aim of this study is to identify more phytochemical compounds in the *C. mas *fruit, particularly bioactive iridoids, by using ultra performance liquid chromatography (UPLC) coupled with photodiode array spectrophotometry (PDA) and electrospray time-of-flight mass spectrometry (ESI-TOF-MS). 

## 2. Materials and Methods

### 2.1. Chemicals and Standards

Optima acetonitrile (MeCN, lot no. 125783), methanol (MeOH, lot no. 124876), and formic acid of LC-MS grade were purchased from Fisher Scientific Co. (Fair Lawn, NJ, USA). Chromasolv^®^ Water (H_2_O, lot no. SHBB9224V) of LC-MS grade was purchased from Sigma-Aldrich (St. Louis, MO, USA). Tartaric acid, malic acid, chlorogenic acid, gallic acid, and rutin standards were purchased from Sigma-Aldrich (St. Louis, MO, USA). Citric acid was purchased from Fisher Scientific Co. Loganic acid was obtained from ChromaDex (Irvine, CA, USA). Loganin, sweroside, and cornuside were purchased from Chengdu Biopurify Phytochemicals Ltd (Sichuan, China). The purities of all standards were higher than 98%. The standards were accurately weighed and then dissolved in an appropriate volume of MeOH to produce corresponding stock standard solutions. Working standard solutions were prepared by diluting the stock solutions with MeOH at different concentrations. All stock and working solutions were maintained at 0°C in a freezer. 

### 2.2. Sample Collection and Preparation

*C. mas* fruit samples were collected from wild trees in the mountains near Kastamonu, Turkey, in 2012. A voucher specimen is deposited in Research and Development Department laboratory of Morinda Inc. (American Fork, Utah, USA). The raw fruits were mashed into puree and the seeds were removed. One gram of mashed fruit puree was weighed accurately and 9 mL of 50% MeOH in H_2_O was added. The mixture was sonicated for 30 minutes and then centrifuged. The supernatant was transferred into a volumetric flask and volume brought to 10 mL with 50% MeOH in H_2_O. All samples were filtered through a nylon microfilter (0.45 *μ*m pore size) before UPLC analysis.

### 2.3. Instrumentation and Chromatographic Conditions

Analyses were performed with an Agilent 1260 Infinity LC System coupled to an Agilent 6230 time-of-flight (TOF) LC/MS System (Agilent Technologies, Santa Clara, CA). The Agilent 1260 LC module was coupled with a photodiode array (PDA) detector and a 6230 time-of-flight MS detector, along with a binary solvent pump and an autosampler. Chromatographic separations were performed with an Atlantis reverse phase C18 column (4.6 mm × 250 mm; 5 *μ*m, Waters Corporation, Milford, MA, USA). The pump was connected to a gradient binary solvent system: A, 0.1% formic acid in H_2_O (v/v) and B, 0.1% formic acid in MeCN. The mobile phase was programmed consecutively in linear gradients as follows: 0–5 min, 98% A and 2% B; 40 min, 70% A and 30% B; 46–52 min, 2% A and 98% B; and 53–55 min, 98% A and 2% B. The ionization source was Agilent Jet Stream, with electrospray ionization (ESI) negative mode employed for acquisition of mass spectra. The elution was run at a flow rate of 0.8 mL/min. UV spectra were monitored in the range of 200 nm and 400 nm. Injection volume was 2 *μ*L for each of the sample solutions, followed by needle wash. The column temperature was maintained at 40°C. Nitrogen, supplied by a nitrogen generator (model no. NM32LA, Peak Scientific Instruments Ltd, Scotland, UK), was used as the drying and nebulizer gas. Other MS instrumental conditions are summarized as follows: drying gas temperature and flow rate were 350°C and 11.0 L/min, respectively; nebulizer pressure was 50 psi; sheath gas temperature and flow rate were 350°C and 12.0 L/min, respectively; capillary, nozzle, and fragmentor voltages were 3500, 500, and 100 V, respectively; skimmer was 65.0; Oct 1 RF *V*_*pp*_ was 750. The instrument state was set to high resolution mode (4 GHz). Tuning and calibration were performed before sample runs. Data collection and integration were performed using MassHunter workstation software (version B.05.00). The data was collected in the range of 100 and 1700 *m*/*z*. 

### 2.4. Accurate Mass Measurement

Data were stored in both centroid and profile formats during acquisition. Two independent reference lock-mass ions, purine (*m*/*z* 119.03632) and HP-0921 (*m*/*z* 966.000725), were employed to ensure mass accuracy and reproducibility. 

### 2.5. Characterization of Phytochemicals

Identification of phytochemical compounds **1–10** was accomplished by elucidating mass spectral data. Compound identities were then confirmed by comparing the UPLC retention times and UV spectra of target peaks with those of reference compounds. 

### 2.6. Primary DNA Damage Test in *E. coli* PQ37

The SOS chromotest in *E. coli* PQ37 was used to determine the potential for loganic acid, loganin, sweroside, and cornuside to induce primary DNA damage. This test was carried out according to a previously developed method [23]. *E. coli* PQ37 was incubated in LB medium in a 96-well plate at 37°C in the presence of the iridoids for 2 hours. The concentrations tested were 7.81, 15.6, 31.2, 62.5, 125, 250, 500, and 1000 *μ*g mL^−1^. Following incubation with replicate samples, 5-bromo-4-chloro-3-indolyl-*β*-D-galactopyranoside was added to the wells to detect *β*-galactosidase enzyme activity, which is induced during SOS repair of damaged DNA. Nitrophenyl phosphate is also added to the wells to measure alkaline phosphatase activity, an indicator of cell viability. The samples were again incubated and the absorbance of each sample, blanks, and controls was measured at 410 and 620 nm with a microplate reader. Vehicle blanks and positive controls, 1.25 *μ*g mL^−1^ 4-nitroquinoline 1-oxide (4NQO), were included in this test. The induction factor of each material was calculated by dividing the absorbance of the sample at 620 nm by that of the blank, while also correcting for cell viability. Induction factors less than two indicate an absence of genotoxic activity.

### 2.7. Antigenotoxicity Test in *E. coli* PQ37

The primary DNA damage test was performed again, similar to the method described above. However, the method was modified to include incubation of *E. coli* PQ37 in the presence of 1.25 *μ*g mL^−1^ 4NQO with 250 *μ*g mL^−1^ loganic acid, loganin, sweroside, or cornuside. Induction factors were calculated in the same manner as described above. The percent reduction in genotoxicity was determined by dividing the difference between the induction factor of 4NQO and the blank (induction factor of 1) by the difference between the induction factor of 4NQO plus iridoid sample and the blank. Experiments were performed in triplicate.

## 3. Results and Discussion

Although the application of LC-MS may minimize the need for chromatographic separation, a good LC separation may prevent ion suppression and isobaric interferences in the MS analysis. The UPLC system delivered a more rapid and effective separation than HPLC, especially for the wide range of phytochemicals identified. UPLC also facilitated the simultaneous determination of the phytochemical matrix of our samples. The flow rate, solvent system, and column were optimized for the UPLC system relative to the analysis of phytochemicals in *C. mas* fruit samples. A moderate flow rate of 0.8 mL/min accommodated electrospray source, column separation and optimized sensitivity and resolution. Better peak shape and resolution for each target peaks, as well as optimal sensitivity for ion detection, were obtained by adding 0.1% formic acid. The column temperature was kept at 40°C to minimize column pressure due to flow rate. A gradient solvent system of MeCN-H_2_O ensured complete separation of target peaks within a limited time frame. The UPLC system delivered good separation and eliminated the overlap of targeted phytochemical peaks.

The time-of-flight mass spectral detector is known for providing accurate and precise mass information. It can accurately measure mass values with a mass error less than 5 ppm. The generation of empirical formulae results in possible identification of phytochemicals by means of elemental composition analysis. Our experiments used electrospray ionization and were operated in negative mode as the interface since it generated higher signal information for the compounds with less interruption compared to positive mode. All other mass spectral parameters, including drying gas flow and temperature, nebulizer pressure, and sheath gas flow and temperature, were also optimized in order to achieve good sensitivity and resolution. 

**Figure 1:**
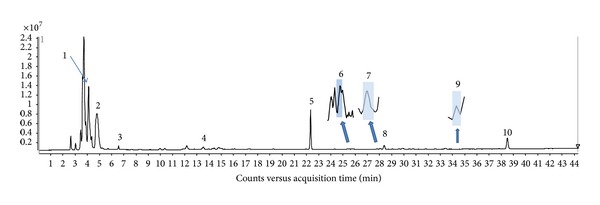
The typical total ion chromatogram (TIC) of *C. mas* fruit puree. Ionization source performed on Agilent Jet Stream TOF-MS with electrospray ionization (ESI) negative mode. (1) *x*-axis represents retention time and *y*-axis is intensity of *m*/*z* peaks. (2) Peaks 1–10 were identified as tartaric acid (**1**), malic acid (**2**), citric acid (**3**), gallic acid (**4**), chlorogenic acid (**6**), rutin (**9**), and four iridoids, loganic acid (**5**), loganin (**7**), sweroside (**8**), and cornuside (**10**). (3) Peaks 6, 7, and 9 were enlarged by zooming in.

The total ion chromatogram (TIC) of *C. mas* fruit is shown in Figure 1. Negative ion electrospray LC-MS chromatograms of the fruit puree resulted in the identification of 10 peaks. Deprotonated molecular ions [M − H]^−^, chlorinated ions [M + Cl]^−^, and/or formic acid adducts [M + HCOO]^−^ of the compounds were identified with the TOF detector. As a result, accurate molecular weights were determined. Since peaks 6, 7, and 9 were not clearly visible on the TIC chromatogram due to their low concentration, zoomed-in peaks are provided accordingly. 

**Figure 2:**
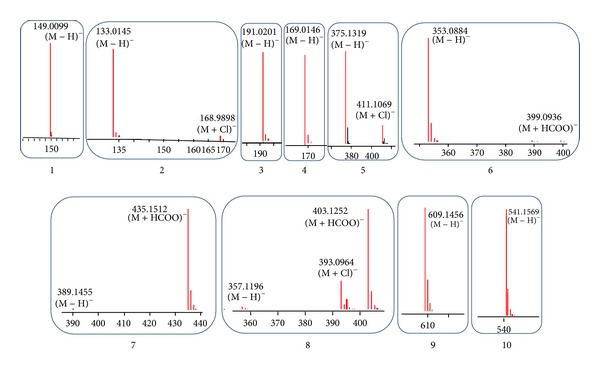
Accurate mass spectra (mass-to-charge, *m*/*z*), in ESI negative mode, of phytochemical peaks identified in *C. mas* fruit by Agilent TOF detector. (1) Numbers 1–10 represent peaks to be characterized, that is, tartaric acid (**1**), malic aicd (**2**), citric acid (**3**), gallic acid (**4**), chlorogenic acid (**6**), a flavonoid glycoside rutin (**9**), and four iridoids, loganic acid (**5**), loganin (**7**), sweroside (**8**), and cornuside (**10**). (2) Accurate mass-to-charge (*m*/*z*) is labeled on the top of each peak.

Accurate mass spectra (mass-to-charge, *m*/*z*) of the phytochemical peaks identified are shown in Figure 2. Peaks 1–4 were compounds that eluted early from the column. Peaks 1, 3, and 4 had deprotonated molecular ions [M − H]^−^ with *m*/*z* values of 149.0099, 191.0201, and 169.0146, respectively, corresponding to the molecular formula of C_4_H_6_O_6_, C_6_H_8_O_7_, and C_7_H_6_O_5_. Peak 2 had [M − H]^−^ and [M + Cl]^−^ with *m*/*z* values of 133.0145 and 168.9898, respectively, matching the molecular formula C_4_H_6_O_5_. Compound 6 had [*M* − *H*]^−^ and [M + HCOO]^−^ at *m*/*z* 353.0884 and 399.0936, corresponding to C_16_H_24_O_10_. Comparisons against reference compound retention revealed that peaks 1–4 and 6 were organic acids, namely, tartaric acid (**1**), malic acid (**2**), citric acid (**3**), gallic acid (**4**), and chlorogenic acid (**6**). By the same methods, peak 9 was determined to be rutin (**9**), a flavonoid glycoside. 

**Figure 3:**
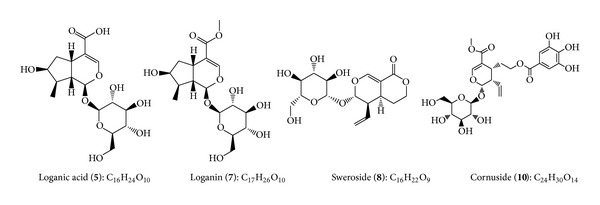
Chemical structures of iridoids identified in *C. mas* fruit by UPLC-TOF-MS.

Peak 5 is a major peak on the chromatogram, eluting at 22.345 min and displaying both [M − H]^−^ and [M + Cl]^−^ at *m*/*z* 375.1319 and 411.1069. Its molecular formula was determined to be C_16_H_24_O_10_. Peak 7 showed [M − H]^−^ and [M + HCOO]^−^ at *m*/*z* 389.1455 and 435.1512. Peak 8 exhibited a deprotonated *m*/*z*, a chlorinated *m*/*z*, and a formic acid adduct of 357.1196, 393.0964, and 403.1252, respectively. Peak 10 had deprotonated ions with *m*/*z* value of 541.1569. By comparing these pieces of information with UV and mass spectra, as well as retention times of reference compounds, peaks 5, 7, 8, and 10 were identified as the iridoids loganic acid (**5**), loganin (**7**), sweroside (**8**), and cornuside (**10**). A summary of the chromatographic and mass spectral data of the phytochemical compounds identified in *C. mas* fruit is given in Table 1. Chemical structures of the iridoids in *C. mas* fruit are provided in Figure 3.

These iridoids have displayed many potential biological activities. Loganic acid has potent anti-inflammatory activity [24]. Loganin and sweroside were observed to have strong antibacterial, antifungal, and antispasmodic activities [25]. Loganin was also found to have neuroprotective effects [26]. Cornuside has remarkable antioxidant activity and may provide protection against acute myocardial ischemia and reperfusion injury [27].

In the primary DNA damage test in *E. coli* PQ37 (Table 2), the mean induction factors for loganic acid, loganin, sweroside, and cornuside, at 1000 *μ*g mL^−1^, were 1.095, 0.995, 1.028, and 1.068, respectively. At all concentrations tested, these iridoids did not induce any SOS repair at a frequency significantly above that of the blank. Statistically, induction factors were not different from that of the blank, and all results remained well below the twofold criteria for genotoxicity. SOS chromotest results have a high level of agreement (86%) with those from the reverse mutation assay [28]. Therefore, the SOS chromotest has some utility in predicting potential mutagenicity, in addition to primary DNA damage. These results are consistent with previously published genotoxicity tests of other food derived iridoids [29]. 

In the antigenotoxicity test, 4NQO exhibited obvious genotoxicity, inducing SOS repair more than 3-fold above that of the vehicle blank. Incubation with loganic acid did not significantly reduce 4NQO induction factors. However, loganin, sweroside, and cornuside apparently reduced the amount of 4NQO that caused DNA damage, as mean induction factors were lowered to below 3 in cells incubated in the presence of these iridoids (Table 3). 

## 4. Conclusions

The UPLC-TOF-MS method reported has been shown to be useful for secondary metabolite profiling of *Cornus mas* fruit. With this method, a total of ten phytochemicals were identified, including five organic acids and four iridoids. To our knowledge, this is the first time that **1–4** and **6–10** have been identified in *C. mas* fruit. Loganin, sweroside, cornuside, and loganic acid will provide valuable information for chemical taxonomy. Iridoids, together with other characteristic components, can be used for identification, authentication, and standardization of *C. mas* fruit raw materials and commercial products. The iridoids in the fruit did not display any toxicity. On the other hand, these iridoids exhibited potential anti-genotoxic activity. Our findings also warrant future research on the safety and efficacy of *C. mas*. 

## Figures and Tables

**Table 1:** Chromatographic and mass spectral data of phytochemical compounds identified in *C. mas* fruits.

Number	Name	Formula	RT	*m/z *	Mass		Score	Area
1	Tartaric acid	C_4_H_6_O_6_	4.084	149.0099	150.0171	[M − H]^−^	96.91	26316227
2	Malic acid	C_4_H_6_O_5_	4.863	133.0145, 168.9898	134.0218	[M − H]^−^ [M + Cl]^−^	99.32	28327606
3	Citric acid	C_6_H_8_O_7_	6.57	191.0201	192.0273	[M − H]^−^	98.81	4041717
4	Gallic acid	C_7_H_6_O_5_	13.529	169.0146	170.0219	[M − H]^−^	85.94	4663081
5	Loganic acid	C_16_H_24_O_10_	22.345	375.1319, 411.1069	376.1372	[M − H]^−^ [M + Cl]^−^	98.79	3904847
6	Chlorogenic acid	C_16_H_18_O_9_	25.725	353.0884	354.0956	[M − H]^−^	98.43	575436
7	Loganin	C_17_H_26_O_10_	28.028	389.1455, 435.1512	390.153	[M − H]^−^ [M + HCOO]^−^	98.79	954718
8	Sweroside	C_16_H_22_O_9_	28.393	357.1196, 393.0964, 403.1252	358.127	[M − H]^−^ [M + Cl]^−^ [M + HCOO]^−^	98.84	3613261
9	Rutin	C_27_H_30_O_16_	34.772	609.1456	610.1532	[M − H]^−^	98.25	28498
10	Cornuside	C_24_H_30_O_14_	38.501	541.1569	542.1641	[M − H]^−^	93.36	17943814

**Table 2:** Primary DNA damage assay in *E. coli* PQ37.

Compound	Concentration (*µ*g mL^−1^)	Induction factor (mean ± standard deviation)
Loganic acid	1000	1.095 ± 0.004
	500	1.080 ± 0.038
	250	0.994 ± 0.003
	125	1.058 ± 0.025
	62.5	1.006 ± 0.024
	31.2	1.027 ± 0.003
	15.6	1.012 ± 0.003
	7.81	0.965 ± 0.031

Loganin	1000	0.995 ± 0.044
	500	1.013 ± 0.029
	250	1.036 ± 0.018
	125	1.051 ± 0.038
	62.5	1.029 ± 0.001
	31.2	1.041 ± 0.001
	15.6	1.033 ± 0.006
	7.81	1.016 ± 0.036

Sweroside	1000	1.028 ± 0.008
	500	1.021 ± 0.005
	250	1.032 ± 0.008
	125	1.043 ± 0.013
	62.5	1.009 ± 0.042
	31.2	1.001 ± 0.010
	15.6	1.029 ± 0.017
	7.81	0.939 ± 0.022

Cornuside	1000	1.068 ± 0.015
	500	1.030 ± 0.018
	250	1.012 ± 0.000
	125	1.074 ± 0.040
	62.5	1.023 ± 0.030
	31.2	1.026 ± 0.016
	15.6	1.019 ± 0.029
	7.81	0.959 ± 0.019

4NQO	1.25	3.365 ± 0.516

**Table 3:** Antigenotoxicity test in *E. coli* PQ37.

Compound	Concentration (*µ*g mL^−1^)	Induction factor (mean ± standard deviation)
4NQO	1.25	3.365 ± 0.516
4NQO + loganic acid	250*	3.278 ± 0.260
4NQO + loganin	250*	2.497 ± 0.821
4NQO + sweroside	250*	2.259 ± 0.325
4NQO + cornuside	250*	2.587 ± 0.199

*Iridoid concentration; 4NQO concentration is 1.25 *µ*g mL^−1^.
